# Ecological and engineered modulation of the mosquito microbiome: mechanisms, vector competence, and translational prospects for disease control

**DOI:** 10.3389/fmicb.2026.1884326

**Published:** 2026-06-26

**Authors:** Ashraf Akintayo Akintola, Lateef Adegboyega Sulaimon, Kamoru Abdulazeez Adeniyi, Ui Wook Hwang

**Affiliations:** 1 Department of Biology, Teachers College and Institute for Phylogenomics and Evolution, Kyungpook National University, Daegu, Republic of Korea; 2 Department of Chemical Science, College of Natural Science, Crescent University, Abeokuta, Nigeria; 3 Department of Biological Sciences, Federal University of Dutse, Dutse, Nigeria; 4 Department of Biomedical Convergence Science and Technology, School of Industrial Technology Advances, Kyungpook National University, Daegu, Republic of Korea; 5 Department of Advanced Bioconvergence, Kyungpook National University, Daegu, Republic of Korea; 6 Institute for Korean Herb-Bio Convergence Promotion, Kyungpook National University, Daegu, Republic of Korea; 7 Phylomics Inc., Daegu, Republic of Korea

**Keywords:** arbovirus, integrated vector management, microbiome modulation, microbiota engineering, mosquito microbiome, paratransgenesis, plasmodium, vector competence

## Abstract

Malaria, dengue fever, Zika, chikungunya, yellow fever, and West Nile fever are mosquito-borne diseases that collectively impose an enormous global health burden, disproportionately affecting low- and middle-income countries where vector-control tools remain limited or compromised by insecticide resistance. Over the past two decades, the characterization of mosquito-associated microbiomes has transformed our understanding of vector biology, revealing complex, ecologically contingent assemblages of bacteria, fungi, viruses, and protists that profoundly influence mosquito physiology, immunity, and pathogen transmission competence. This review synthesizes current knowledge on the composition and determinants of the mosquito microbiome across major vector genera—Aedes, Anopheles, and Culex—and critically evaluates evidence for microbiome roles in larval development, adult fitness, immune homeostasis, and pathogen–vector interactions. We examine how resident microbiota can inhibit or, in some contexts, facilitate pathogen establishment, dissemination, and transmission, and we discuss the mechanistic pathways underlying these effects, including immune priming, niche competition, antimicrobial metabolite production, and modulation of midgut barrier integrity. We then review major strategies for deliberate microbiome modulation, including *Wolbachia*-based pathogen blocking and population suppression, paratransgenesis, symbiont supplementation, microbiota engineering, and habitat-level manipulation, and evaluate their biological rationale, current evidence base, field feasibility, and limitations. Attention is given to the gap between laboratory proof-of-concept and operational deployment, as well as to biosafety, regulatory, ecological, and ethical challenges that must be resolved before microbiome-based interventions can be integrated into public health programs. We conclude by identifying priority research questions and the technological advances most likely to accelerate progress from descriptive microbiome science to predictive, actionable vector control.

## 1 Introduction

Mosquitoes rank among the most medically significant arthropods on Earth. Globally, *Anopheles* mosquitoes transmit *Plasmodium falciparum* and *P. vivax*, the dominant causative agents of malaria, which in 2022 accounted for an estimated 282 million clinical cases and approximately 610,000 deaths, the vast majority in sub-Saharan Africa (World Health Organization, 2025). Simultaneously, *Aedes aegypti* and *Aedes albopictus* serve as the principal vectors of dengue virus (DENV), Zika virus (ZIKV), chikungunya virus (CHIKV), and yellow fever virus (YFV), affecting hundreds of millions of people annually across tropical and subtropical regions. Culex mosquitoes transmit West Nile virus (WNV) and the filarial parasite *Wuchereria bancrofti*, further extending the epidemiological burden of this single insect family (Bhatt et al., 2013; Messina et al., 2019).

Against this background, the mosquito microbiome, known as the totality of microorganisms associated with a mosquito host and its developmental environments, has emerged as a major frontier in vector biology and disease control research. Early descriptive surveys using culture-based techniques identified bacterial associates of mosquitoes, but the advent of culture-independent 16S rRNA amplicon sequencing and, subsequently, shotgun metagenomics, has revealed microbiome diversity far exceeding earlier estimates and has demonstrated that these communities are dynamic, spatially structured, and ecologically context-dependent (Minard et al., 2013; Osei-Poku et al., 2012; Wang et al., 2023). Crucially, accumulating evidence demonstrates that microbiome composition is not epidemiologically neutral. Resident microbiota can substantially alter the capacity of a mosquito to acquire, develop, and transmit pathogens. This property is referred to as vector competence (Weiss and Aksoy, 2011; Gendrin and Christophides, 2013; Palinauskas et al., 2024).

**Figure 1:**
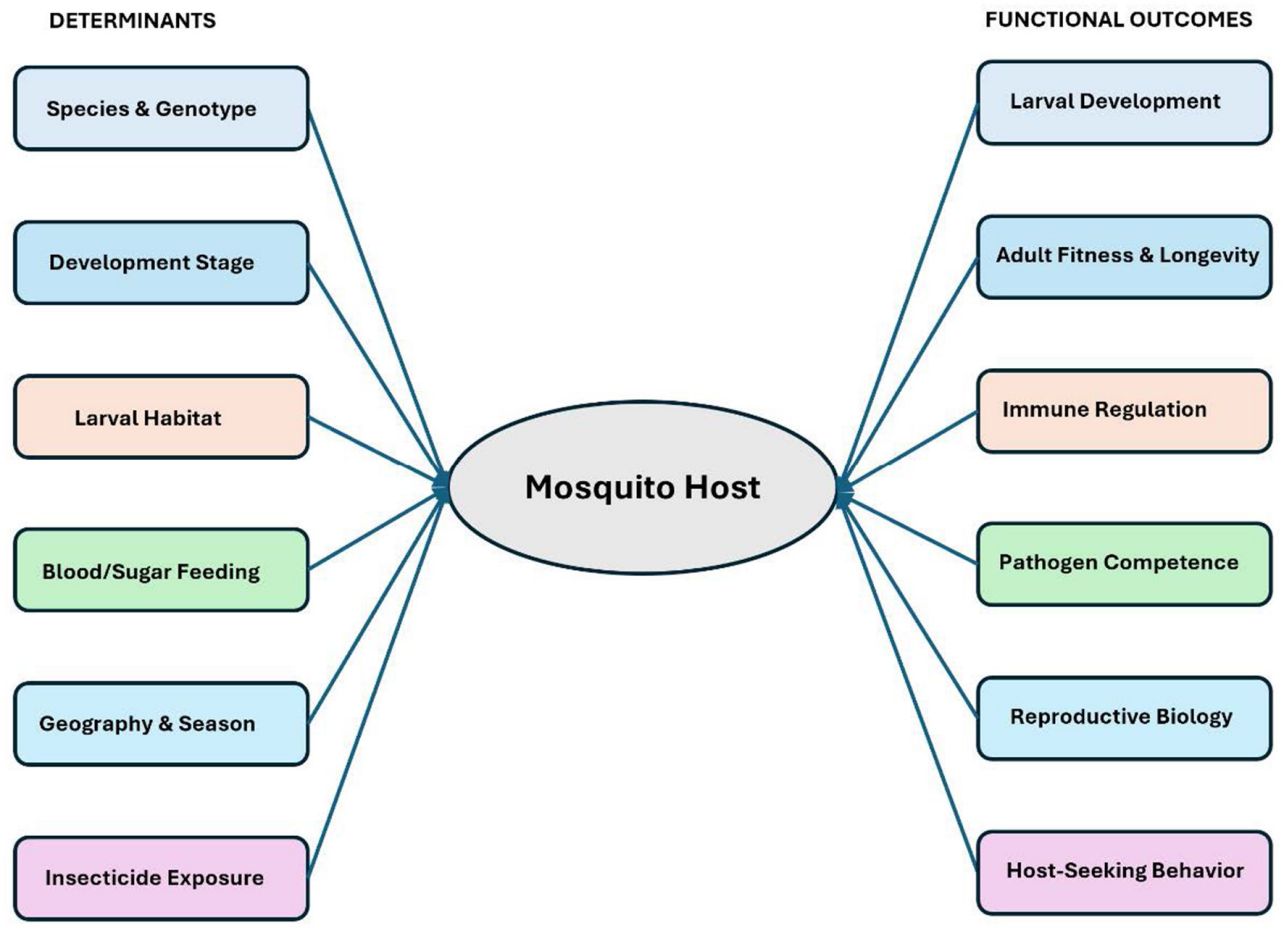
Determinants and functional outcomes of the mosquito microbiome. The microbiome is shaped by an interacting set of host and environmental factors (**left panel**) and in turn influences key mosquito biological traits with direct relevance to vector competence and disease transmission (**right panel**). Arrows indicate directionality of influence; outcomes are not uniformly positive or negative from a disease-control perspective. Matching colors between determinant categories (**left panel**) and outcome categories (**right panel**) do not imply exclusive or direct causal associations. Each determinant influences multiple outcomes and each outcome is shaped by multiple determinants. Colors are used for visual differentiation within each panel only and should not be interpreted as indicating paired relationships across panels.

The concept of “microbiome modulation” in mosquito research encompasses both the ecological processes that naturally shape microbial communities and deliberate interventions designed to alter those communities for disease-control purposes. Natural modulation arises from environmental factors such as larval habitat chemistry, host plant associations, geographical location, developmental stage, blood-meal source, and insecticide exposure, that continuously restructure microbiome composition (Figure 1). Deliberate modulation encompasses strategies ranging from the introduction of maternally inherited endosymbionts such as *Wolbachia* to sophisticated synthetic-biology approaches that engineer bacterial symbionts to express pathogen-inhibiting molecules. This review addresses these dimensions systematically, proceeding from fundamental microbiome biology to translational disease-control applications. Our central argument is that the mosquito microbiome represents a genuinely promising but incompletely understood target for next-generation vector control, requiring rigorous mechanistic research, expanded field validation, and careful governance frameworks. Crucially, most microbiome strategies remain at early laboratory or proof-of-concept phases, with *Wolbachia* wMel in *Ae. aegypti* as the sole strategy to have achieved randomized controlled trial-level field evidence—a translational gap that frames the critical evaluation in this review.

## 2 Composition and determinants of the mosquito microbiome

### 2.1 Taxonomic overview

The mosquito microbiome is taxonomically diverse, encompassing bacteria, fungi, viruses, and unicellular eukaryotes. Bacterial communities have been the most extensively characterized fraction. Across *Aedes, Anopheles*, and *Culex* species, phyla-level surveys consistently identify Proteobacteria (particularly Alphaproteobacteria and Gammaproteobacteria), Firmicutes, Actinobacteria, and Bacteroidetes as dominant groups, although relative abundances vary substantially among studies, geographic regions, and host species (Minard et al., 2013; Terenius et al., 2012; Ragini et al., 2025). Genera repeatedly identified include *Asaia, Serratia, Elizabethkingia, Enterobacter, Pseudomonas*, and *Chromobacterium*, though no single genus is universally dominant (Hegde et al., 2015; Coon et al., 2016; Wang et al., 2023). The endosymbiont *Wolbachia* is naturally absent from major *Anopheles* populations and most *Ae. aegypti* field populations but prevalent in many *Culex quinquefasciatus* populations (Hilgenboecker et al., 2008).

#### 2.1.1 Fungal microbiome (mycobiome)

The mosquito mycobiome has received substantially less attention than the bacterial fraction, but is increasingly recognized as a functional contributor to host biology. Yeast-like fungi in the genera *Candida, Trichosporon*, and *Debaryomyces* have been recovered from mosquito larvae and adults. Hegde et al. (2024) conducted the first systematic mycobiome comparison across *Ae. albopictus, Ae. aegypti*, and *Cx. quinquefasciatus* using ITS amplicon sequencing and found that both host species identity and environmental origin significantly shaped fungal community diversity, and that inter-kingdom interactions between bacterial and fungal communities modulate overall microbiome structure. Entomopathogenic fungi—including *Metarhizium* and *Beauveria* species—have been recovered from mosquito midguts and represent biologically significant eukaryotic microbiome constituents: *beauveria bassiana* and *Metarhizium anisopliae* actively reduce adult mosquito survival and fecundity, providing a direct link between the mycobiome and vector population dynamics (Scholte et al., 2004; Hegde et al., 2024).

#### 2.1.2 Protozoan, nematode, and broader eukaryotic microbiome components

Beyond bacteria and fungi, the mosquito eukaryotic microbiome includes protozoan microorganisms and nematode-associated organisms that influence host biology and vector competence. Gregarine apicomplexan parasites (e.g., *Ascogregarina* spp.) colonize *Aedes* midguts and have been shown to reduce larval survival and alter adult body size in ways that indirectly affect vectorial capacity. *Mermithidae* nematodes are naturally occurring larval parasites of *Anopheles* and *Culex* that can substantially reduce larval survival and thus suppress vector populations. While mechanistic data on protozoan and nematode contributions to vector competence remain limited, the application of shotgun metagenomics now enables simultaneous profiling of all kingdoms of the microbiome and is beginning to reveal eukaryotic diversity previously invisible to 16S rRNA-based approaches (Ragini et al., 2025; Wang et al., 2023).

#### 2.1.3 The virome

The virome, which encompasses insect-specific flaviviruses, rhabdoviruses, phleboviruses, and other RNA viruses, has received substantial and accelerating research attention, particularly following metagenomic discoveries of diverse mosquito-restricted viral lineages (Bolling et al., 2012; Blitvich and Firth, 2015). Recent work has revealed that insect-specific viruses can modulate vector competence through mechanisms including superinfection exclusion and immune pathway modulation (Palinauskas et al., 2024; Ragini et al., 2025).

### 2.2 Tissue-specific communities

Microbial distributions within mosquitoes are spatially heterogeneous as shown in Table 1. The midgut harbors the most abundant and diverse communities, influenced by blood-meal processing, immune activation, and digestive enzyme secretion (Gendrin et al., 2015). Salivary glands carry distinct assemblages, often with lower diversity but greater functional implications for pathogen transmission, since salivary gland invasion is a prerequisite for delivery of both arboviruses and malaria parasites to the vertebrate host during blood feeding (Schneider et al., 2021). Reproductive tissues harbor endosymbionts, including *Asaia* and *Wolbachia*, which exploit vertical transmission and persist across generations (Damiani et al., 2010). The larval aquatic environment constitutes a distinct microbiome reservoir: larvae filter-feed on microbial biofilms, and the composition of breeding-site microbiota is a primary determinant of the communities later recovered in adults (Muturi et al., 2017; Coon et al., 2014; Otani et al., 2025).

**Table 1:** Principal microbial taxa associated with major mosquito vector genera and their documented functional roles.

Microbial taxon	Mosquito genus	Tissue location	Functional role/effect	Key references
*Wolbachia* (wMel, wAlbB)	*Aedes*	Intracellular (all tissues)	Pathogen blocking (DENV, ZIKV, CHIKV); CI-mediated population. suppression	Moreira et al., 2009; Utarini et al., 2021
*Serratia marcescens*	*Anopheles, Aedes*	Midgut, hemocoel, salivary glands	Reduces *Plasmodium* oocyst burden; produces serrawettin antimicrobials	Dong et al., 2009; Wang et al., 2023
*Enterobacter* (Esp_Z)	*Anopheles*	Midgut	ROS-mediated killing of *Plasmodium* ookinetes	Cirimotich et al., 2011
*Chromobacterium* spp.	*Anopheles*	Midgut, larval habitat	Romidepsin production inhibits *Plasmodium* development	Saraiva et al., 2018
*Asaia* spp.	*Anopheles, Aedes*	Midgut, salivary glands, reproductive organs	Vertical/venereal transmission; paratransgenesis vehicle; fitness effects variable	Damiani et al., 2010
*Elizabethkingia anophelis*	*Anopheles*	Midgut, salivary glands	Common field isolate; effects on *Plasmodium* poorly characterized	Terenius et al., 2012
Insect-specific flaviviruses	*Culex, Aedes*	Systemic (all tissues)	Superinfection exclusion against related arboviruses (WNV, DENV)	Bolling et al., 2012
*Bacillus thuringiensis israelensis*	*Anopheles, Culex* (larval)	Larval midgut	Larvicidal via Cry toxins; disrupts larval aquatic microbiome	Berry, 2012
*Pseudomonas* spp.	*Culex, Anopheles*	Midgut	Nutrient cycling; immunostimulatory effects; variable pathogen interactions	Minard et al., 2013
*Metarhizium*/*Beauveria* spp. (fungi)	*Aedes, Anopheles, Culex*	Midgut, cuticle	Entomopathogenic; reduces adult survival and fecundity; potential biocontrol tool	Scholte et al., 2004; Hegde et al., 2024

CI, cytoplasmic incompatibility; DENV, dengue virus; ZIKV, Zika virus; CHIKV, chikungunya virus; WNV, West Nile virus; ROS, reactive oxygen species.

### 2.3 Determinants of microbiome structure

A consistent finding across studies is that the mosquito microbiome is highly context-dependent, shaped by an interacting set of host and environmental variables. Host species identity exerts a strong influence: phylogenetically controlled comparisons demonstrate that *Anopheles gambiae* and *Ae. aegypti* harbor compositionally distinct communities even when reared in shared laboratory conditions (Osei-Poku et al., 2012). Developmental stage imposes dramatic restructuring, with pupation representing a major bottleneck at which community diversity contracts (Wang et al., 2011). Blood feeding drives rapid shifts in midgut bacterial abundance and diversity (Gendrin et al., 2015). Insecticide exposure alters gut microbiome composition, potentially through selection of tolerant microorganisms or changes in host immune regulation (Dada et al., 2018). Geographic location and larval habitat chemistry are also major determinants, as recently confirmed in multi-site studies across Korea and Europe (Lee et al., 2024; Otani et al., 2025).

## 3 Functional roles of the mosquito microbiome

### 3.1 Larval development and nutrition

Some of the strongest functional evidence for the mosquito microbiome concerns its necessity for normal larval development. Coon et al. (2014) demonstrated, using germ-free larval rearing systems, that axenic *Anopheles gambiae* larvae fail to develop beyond early instars unless supplemented with bacteria, pointing to an obligatory relationship between gut microbiota and developmental progression. Subsequent work identified bacterial contributions to larval nutrition through nitrogen fixation, B-vitamin synthesis, and processing of dietary carbon sources in nutrient-poor aquatic environments (Strand, 2018; Buck et al., 2016; Coon et al., 2016).

### 3.2 Adult fitness, longevity, and fecundity

In adult mosquitoes, microbiome associations with fitness parameters—including longevity, flight ability, and reproductive output—have been documented. Germ-free adult *Ae. aegypti* exhibit altered fecundity and lifespan trajectories relative to conventionally colonized counterparts, suggesting that gut bacteria contribute to metabolic homeostasis and reproductive physiology (Xi et al., 2008). Some bacteria supplement essential amino acids and vitamins limiting in sugar-only diets, of particular relevance for male mosquitoes that never take blood meals (Strand, 2018; Coon et al., 2016).

### 3.3 Immune regulation and priming

Resident microbiota interact extensively with mosquito innate immunity. The Toll pathway, immune deficiency (Imd) pathway, and JAK-STAT pathway are tonically activated by gut microbiota under homeostatic conditions, and this baseline activation can confer protection against pathogen challenge through immune priming (Dong et al., 2009; Gendrin and Christophides, 2013; Xi et al., 2008). Antibiotic elimination of gut bacteria has been shown to render mosquitoes more susceptible to *Plasmodium* infection in some experimental systems, implicating baseline microbiota-driven immune activation in anti-parasite defense (Dong et al., 2009). This effect is not universal as outcomes depend on the specific bacteria, parasite isolate, mosquito strain, and timing of antibiotic treatment.

### 3.4 Behavior and physiological performance

Microbiome composition influences mosquito behavior, including host-seeking and feeding activity, through multiple documented mechanisms. Volatile organic compounds (VOCs) produced by gut bacteria are detected by the olfactory system and modify host-attraction behaviors (Verhulst et al., 2011). This phenomenon is bidirectional: the human skin microbiome produces VOCs that determine mosquito host preference, and engineering the skin microbiome to alter VOC profiles represents a novel but early-stage approach to bite prevention (Coutinho-Abreu et al., 2024; Bezerra-Santos et al., 2024). Microbiome effects on redox homeostasis, particularly neutralization of reactive oxygen species generated during blood-meal digestion, have been proposed to influence post-feeding survival and thereby affect the duration of the transmission window (Cirimotich et al., 2011). Distinguishing genuine microbiome-driven behavioral effects from correlational associations in field populations remains methodologically challenging.

## 4 Mosquito–microbiome–pathogen interactions

**Figure 2:**
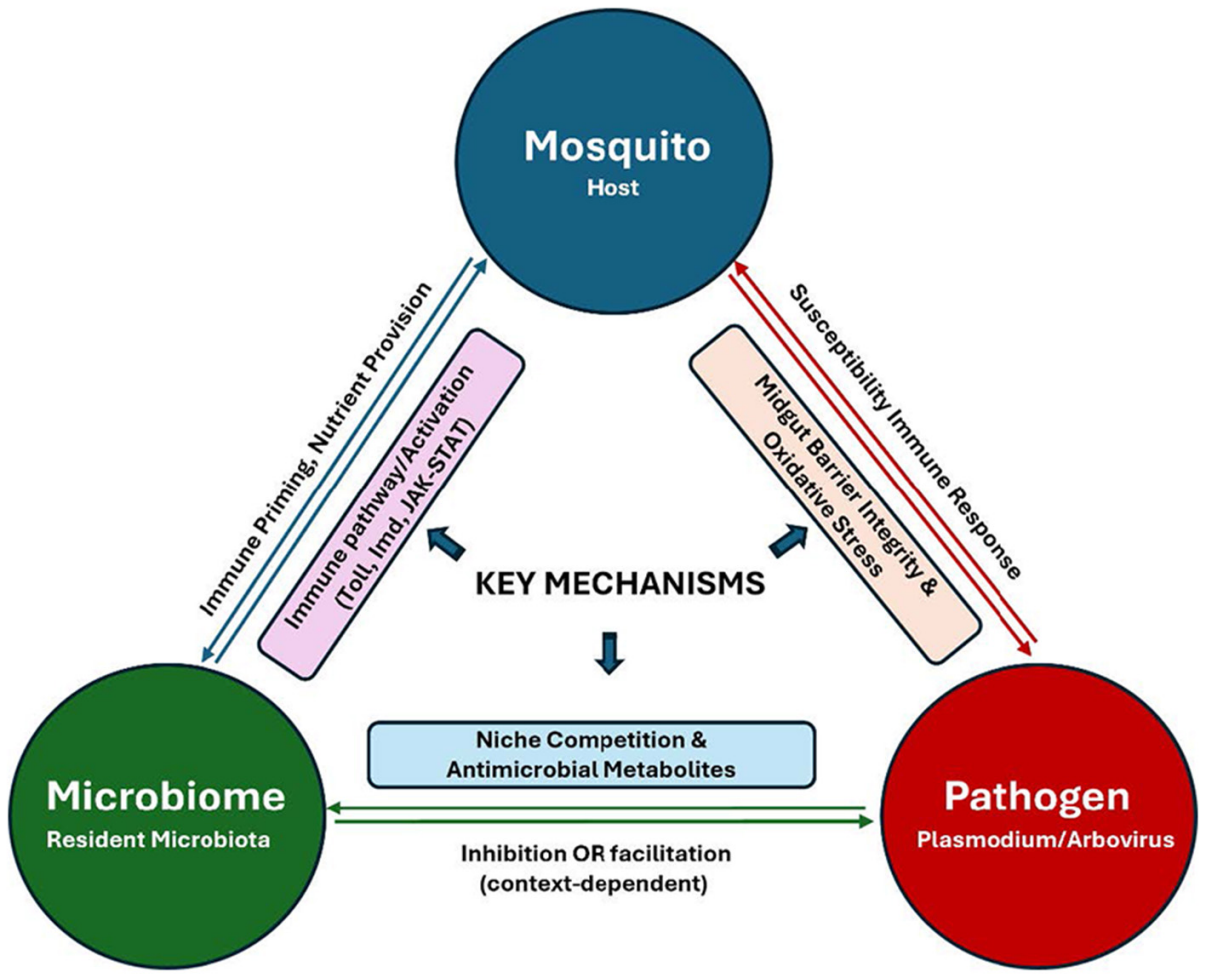
Tripartite interaction network: mosquito host–microbiome–pathogen. Bidirectional arrows represent documented interaction directions. The microbiome can inhibit pathogens through immune priming, niche competition, and antimicrobial metabolite secretion, or, under specific conditions, facilitate infection. Key mechanisms are listed in the lower panel. Arrow weight does not imply quantitative effect size.

The interactions among mosquito host, resident microbiota, and invading pathogens constitute the mechanistic core of this field (Figure 2). These are inherently tripartite and bidirectional: microbiota alter host susceptibility to pathogens; pathogens alter microbiome composition; and the host immune system mediates both sets of interactions in context-dependent ways. Crucially, the net outcome—whether the microbiome functions as a barrier to or a facilitator of pathogen transmission—cannot be inferred from any single component in isolation.

### 4.1 Microbiome interactions with plasmodium

The relationship between Anopheles gut microbiota and Plasmodium development is multifaceted. Boissière et al. (2012) demonstrated that bacterial diversity in *An. gambiae* midguts are inversely associated with *Plasmodium falciparum* oocyst counts in wild-caught mosquitoes, providing correlative evidence that microbiome composition influences parasite transmission potential. Mechanistic studies have identified several pathways by which bacteria antagonize Plasmodium. They include immune priming that activates Toll and Imd pathways to impede ookinete invasion, direct antagonism through secretion of antimicrobial compounds also toxic to parasites, and competition for midgut resources and physical niche exclusion.

*Serratia marcescens*, Enterobacter species (particularly the Esp_Z isolate), and *Chromobacterium* sp. have each been shown experimentally to reduce Plasmodium infection in Anopheles when present in sufficient quantities (Dong et al., 2009; Cirimotich et al., 2011; Saraiva et al., 2018). The strain-specific nature of these effects deserves emphasis. Antagonism depends on metabolic capabilities, including violacein production by Chromobacterium and reactive oxygen intermediate generation by Enterobacter. Not all bacteria inhibit Plasmodium; some are neutral with respect to parasite development, and others suggest potential facilitation under specific conditions. The prevailing laboratory-derived picture must therefore be qualified by the recognition that field microbial communities are compositionally complex, and net outcomes *in vivo* depend on the relative abundances and metabolic activities of multiple microbial constituents simultaneously.

### 4.2 Microbiome interactions with arboviruses

The relationship between mosquito gut microbiota and arboviruses, including DENV, ZIKV, CHIKV, YFV, and WNV, has attracted increasing attention. Dickson et al. (2017) and Tchioffo et al. (2016) demonstrated that bacterial midgut microbiome composition influences dengue virus infection rates in *Ae. aegypti*. Several mechanisms have been proposed: upregulation of antiviral immune pathways (particularly the JAK-STAT pathway); competition for cellular receptors or lipid membrane components required for viral entry; and production of bacterial metabolites that reduce midgut epithelial permeability to viral particles. Insect-specific viruses (ISVs) represent an underexplored dimension of the microbiome. Arbovirus interaction, with several studies demonstrating that ISV-infected mosquitoes show altered susceptibility to related medically important arboviruses - a phenomenon termed superinfection exclusion (Bolling et al., 2012; Blitvich and Firth, 2015; Palinauskas et al., 2024).

#### 4.2.1 Eukaryotic microbiome modulation of arbovirus competence

Beyond bacteria, the eukaryotic microbiome can modulate arbovirus vector competence. Emerging evidence indicates that fungal gut communities interact with mosquito immune pathways-including the Toll and JAK-STAT pathways-and may influence the capacity to transmit dengue and Zika viruses (Palinauskas et al., 2024). Protozoan gut colonizers may similarly modulate immunity in ways that alter viral dissemination rates, though mechanistic data remain limited. The application of kingdom-comprehensive shotgun metagenomics is beginning to resolve these interactions at appropriate resolution (Ragini et al., 2025).

### 4.3 Cases of microbiome-mediated pathogen facilitation

It would be misleading to characterize the microbiome exclusively as a barrier to pathogen establishment. Several studies have identified conditions under which resident microbiota facilitate rather than inhibit infection. Some evidence suggests that certain gut bacteria can enhance Plasmodium sporogony by contributing metabolic precursors required for parasite development, and that experimental elimination of specific commensals can reduce, rather than increase, oocyst burdens. For arboviruses, some evidence suggests that midgut bacteria modulate blood-meal-induced immune suppression in ways that may create a window of opportunity for viral dissemination (Dong et al., 2009). These findings underscore the principle that microbiome–pathogen relationships are not inherently antagonistic, and that the direction of effects is context-dependent.

## 5 Strategies for modulating the mosquito microbiome

### 5.1 Wolbachia-based approaches

**Figure 3:**
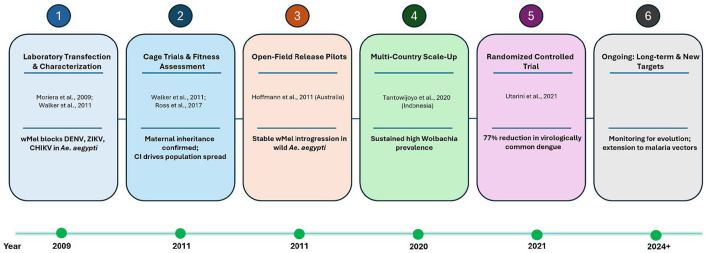
The *Wolbachia* translational pipeline: from laboratory discovery to field deployment. Sequential stages from initial laboratory transinfection (Moreira et al., 2009) through to the randomized controlled trial in Yogyakarta (Utarini et al., 2021), with approximate milestone years indicated on the timeline. Barrier annotations indicate key challenges encountered at each transition. This pipeline illustrates the resource-intensive and multi-decade trajectory required to achieve operational proof-of-efficacy for a microbiome-based intervention.

Among microbiome-based vector-control strategies, the deployment of *Wolbachia* is the most advanced and best validated (Figure 3). *Wolbachia* are maternally inherited intracellular alpha-proteobacteria that naturally infect many insect species, often inducing reproductive phenotypes—including cytoplasmic incompatibility (CI), parthenogenesis, male killing, and feminization—that drive their spread through host populations (Werren et al., 2008). *Wolbachia* was not naturally present in field populations of *Ae. aegypti*, but stable transfection with the wMel strain derived from Drosophila melanogaster has been achieved (Hoffmann et al., 2011). The wMel strain confers substantial inhibition of dengue, Zika, and chikungunya viruses in *Ae. aegypti*, through mechanisms including activation of the JAK-STAT antiviral pathway, competition for intracellular cholesterol required for viral replication, and induction of innate immune priming (Moreira et al., 2009; Walker et al., 2011).

Field deployments of wMel-carrying *Ae. aegypti*, most prominently in Australia, Colombia, Brazil, Indonesia, Vietnam, and several other countries, have shown sustained introgression of *Wolbachia* into wild populations following initial releases (Hoffmann et al., 2011; Tantowijoyo et al., 2020; World Mosquito Program, 2024). A cluster-randomized controlled trial conducted in Yogyakarta, Indonesia, by Utarini et al. (2021) demonstrated a 77% reduction in virologically confirmed dengue incidence in wMel-release areas compared with control areas, representing the most rigorous clinical evidence to date for any microbiome-based vector-control intervention. This trial marked a landmark milestone in translational vector biology.

Despite this substantial success, limitations abound in *Wolbachia*-based strategies. Pathogen-blocking efficacy is strain-specific and can be influenced by mosquito genotype and *Wolbachia* tissue density (Ye et al., 2015). Temperature increases consistent with projected climate change have been shown to reduce wMel density and partially attenuate pathogen blocking (Ross et al., 2017; Frentiu, 2017). *Wolbachia* deployment against *Anopheles* malaria vectors remains at an earlier stage, unlike *Ae. aegypti*, in which stable wMel transinfection is achieved naturally through self-spreading introgression. No analogous maternally transmitted endosymbiont capable of achieving high-frequency population spread has been stably established in wild *An. gambiae* populations to date, representing a key unresolved delivery challenge for microbiome-based malaria control (Bian et al., 2013). Recent advances in wAlbB transinfection of *An. stephensi* represent a promising development, but field-level evidence remains limited (Joshi et al., 2014; Liang et al., 2024).

### 5.2 Paratransgenesis

Paratransgenesis refers to the genetic engineering of commensal or symbiotic microorganisms associated with a vector host to express molecules that interfere with pathogen development, with the modified microbe reintroduced into the vector population. In mosquitoes, the strategy typically involves engineering gut bacteria—including *Serratia, Asaia, Enterobacter*, or *Pantoea agglomerans*—to express single-chain antibodies, lectins, anti-*Plasmodium* peptides, or antiviral RNA molecules (Wilke and Marrelli, 2015; Wang et al., 2012). Wang et al. (2012) demonstrated that *Asaia* engineered to express a single-chain antibody directed against *Plasmodium falciparum* surface protein Pfs25 reduced oocyst numbers in *An. stephensi* under laboratory conditions, providing a compelling proof-of-concept. Paratransgenesis faces substantial challenges, however, including transgene stability over multiple bacterial generations, regulatory hurdles, and ecological persistence of engineered symbionts in field populations (Wilke and Marrelli, 2015).

### 5.3 Symbiont supplementation, microbiota engineering, and ecological approaches

Less technologically intensive than paratransgenesis, supplementation of wild mosquito populations with naturally occurring, pathogen-inhibiting bacteria has been explored as a more ecologically conservative intervention. Field experiments have assessed sugar baits and larval habitat supplementation as vehicles for delivering bacteria, including *Chromobacterium* sp., into *Anopheles* populations, with variable colonization success (Saraiva et al., 2018). The principal advantage is the avoidance of transgenic organisms and their regulatory complexity; the principal limitation is that natural bacteria lack the intrinsic reproductive advantages of maternally transmitted endosymbionts, making population-level persistence problematic without continuous introduction.

Since larval habitat microbiota are primary determinants of adult mosquito microbiomes, manipulation of breeding-site microbial communities offers an ecologically grounded intervention approach. Habitat enrichment with specific organic substrates that favor the growth of pathogen-suppressive bacteria, or removal of organic pollution that favors pathogen-permissive communities, represent potential avenues for community-level microbiome management. The use of *Bacillus thuringiensis israelensis* (Bti) as a larvicide acts partly through direct toxicity but its effects on the broader larval aquatic microbiome and downstream consequences for adult microbial communities have received comparatively little attention (Berry, 2012). Antibiotic perturbation experiments, while informative for demonstrating correlative microbiome–competence associations, should not be interpreted as direct evidence for the specific contributions of particular taxa, given the broad physiological effects of antibiotics on mosquito host tissues (Abdul-Ghani et al., 2012). Table 2 gives a summary of microbiome-pathogen interaction studies in major mosquito vector systems.

**Table 2:** Summary of microbiome–pathogen interaction studies in major mosquito vector systems.

Pathogen	Mosquito Genus	Microbe(s)	Outcome	Mechanism	References
*P. falciparum*	*An. gambiae*	*Enterobacter* Esp_Z	Inhibition (↓ oocysts)	ROS-mediated ookinete killing	Cirimotich et al., 2011
*P. falciparum*	*An. gambiae*	*Serratia marcescens*	Inhibition	Immune priming + antimicrobials	Dong et al., 2009
*P. falciparum*	*An. gambiae*	*Chromobacterium* sp.	Inhibition	Romidepsin; immune upregulation	Saraiva et al., 2018
*P. falciparum*	*An. gambiae*	Diverse field microbiota	Inverse correlation (diversity)	Immune priming (proposed)	Boissière et al., 2012
DENV 1–4	*Ae. aegypti*	Gut microbiota (varied)	Variable; mostly inhibitory	JAK-STAT activation; barrier integrity	Dickson et al., 2017
DENV, ZIKV, CHIKV	*Ae. aegypti*	*Wolbachia* (wMel)	Strong inhibition	Cholesterol competition; JAK-STAT	Moreira et al., 2009
WNV	*Culex* spp.	Culex flavivirus (ISV)	Inhibition (superinfection exclusion)	Viral interference	Bolling et al., 2012
DENV	*Ae. aegypti*	Disrupted microbiome	Facilitation (context-specific)	Loss of immune priming	Dong et al., 2009

DENV, dengue virus; ZIKV, Zika virus; CHIKV, chikungunya virus; WNV, West Nile virus; ISV, insect-specific virus; ROS, reactive oxygen species; ↓ denotes reduction.

## 6 Disease-control applications and translational potential

### 6.1 Reduction of vector competence

The most immediately promising application of microbiome modulation for disease control is the reduction of vector competence through the introduction or enrichment of pathogen-blocking microbial associates. The *Wolbachia* wMel strategy provides the most operationally advanced example, demonstrating that stable, self-spreading microbiome modification can produce epidemiologically significant reductions in dengue incidence at the community level (Utarini et al., 2021). For malaria, direct bacterium-mediated reduction of *Plasmodium* competence has been demonstrated under controlled conditions, but translating this finding to a deployable intervention requires solving the persistence problem that *Wolbachia*-based strategies circumvent through maternal inheritance.

### 6.2 Population suppression

*Wolbachia*-based cytoplasmic incompatibility has been harnessed for population suppression through release of incompatible males. Male-only releases of wAlbB-infected *Ae. albopictus* have demonstrated substantial suppression of target populations in treated areas in China (Zheng et al., 2019). This approach differs conceptually from population replacement: suppression aims to reduce mosquito abundance rather than change microbiome composition of a persistent wild population.

### 6.3 Integration with existing vector-management strategies

Microbiome-based interventions are most plausibly implemented as components of integrated vector management (IVM) programs that also include insecticide-based control, environmental management, biological control, and surveillance (Table 3). The complementarity of microbiome interventions with the sterile insect technique (SIT) is also being explored: combining *Wolbachia*-mediated CI with radiation-induced sterility may enhance the effectiveness of population suppression while reducing required release ratios (Bourtzis et al., 2016). A critical distinction must be maintained between laboratory proof-of-concept, small-scale field demonstrations, and operational implementation. For most microbiome strategies, the evidence progression falls substantially short of the rigorous translational pathway achieved by *Wolbachia*.

**Table 3:** Key microbiome-based vector-control strategies: evidence base, field status, and implementation challenges.

Strategy	Target pathogen(s)	Current stage	Evidence	Key challenges	Leading references
*Wolbachia* pop. replacement (wMel in *Ae. aegypti*)	DENV, ZIKV, CHIKV	RCT/Multi-country deployment	⋆⋆⋆⋆⋆	Thermal sensitivity; evolutionary escape risk	Utarini et al., 2021; Hoffmann et al., 2011
*Wolbachia* pop. suppression (CI-based male release)	Multiple arboviruses	Open-field trials	⋆⋆⋆⋆✰	Male-only sexing; re-invasion; repeated releases	Zheng et al., 2019; Turner, 2024; Shepard et al., 2024; Zimmermann et al., 2024; World Mosquito Program, 2024
*Wolbachia* wAlbB in *An. stephensi* (malaria)	*Plasmodium* spp.	Field cage study	⋆⋆✰✰✰	Stable Anopheles transinfection; no wild-population trial	Joshi et al., 2014; Liang et al., 2024
Paratransgenesis (engineered gut bacteria)	*Plasmodium*, DENV	Laboratory proof-of-concept	⋆⋆✰✰✰	Transgene stability; regulatory approval; ecological persistence	Wang et al., 2012; Wilke and Marrelli, 2015
Symbiont supplementation (natural bacteria)	*Plasmodium*	Lab/limited field trials	⋆⋆✰✰✰	No maternal inheritance; requires repeated delivery	Cirimotich et al., 2011
Insect-specific virus deployment	DENV, WNV	Early laboratory	⋆⋆✰✰✰	Delivery mechanism unknown; host-range uncertainty	Bolling et al., 2012
Larval habitat microbiome manipulation	*Plasmodium*, arboviruses	Observational/conceptual	⋆✰✰✰✰	Ecological complexity; difficult to control *in situ*	Berry, 2012; Muturi et al., 2017

Evidence strength rated on a five-star scale based on study design quality, reproducibility, and field validation. Star ratings indicate the relative strength or level of evidence/assessment used in this study, where ⋆⋆✰✰✰= low, ⋆⋆⋆✰✰= moderate, ⋆⋆⋆⋆✰= high, and ⋆⋆⋆⋆⋆= very high.

CI, cytoplasmic incompatibility; RCT, randomized controlled trial.

### 6.4 Recent field trial updates (2022–2025)

Since the Yogyakarta trial (Utarini et al., 2021), *Wolbachia* wMel releases have been expanded to additional sites in Colombia, Brazil, Fiji, New Caledonia, Kiribati, and Vanuatu, with sustained introgression confirmed in all locations monitored (World Mosquito Program, 2024). For *Anopheles* malaria vectors, a field cage study of wAlbB-infected *An. stephensi* demonstrated stable maternal inheritance and partial *Plasmodium* inhibition in cage conditions (Joshi et al., 2014; Liang et al., 2024), representing the most advanced progress to date for malaria-specific *Wolbachia* deployment. Paratransgenesis approaches have advanced through improved stable expression systems in *Serratia*, but remain at laboratory proof-of-concept level. No new open-field trials of paratransgenesis or bacterial supplementation have been completed since 2021.

## 7 Challenges, constraints, and biosafety considerations

### 7.1 Microbiome instability and poor reproducibility

One of the most significant obstacles in mosquito microbiome research is poor reproducibility across laboratories, geographic settings, and experimental conditions. Studies conducted on the same mosquito species but in different geographic contexts frequently report compositionally distinct microbiomes at the genus and species level, and even within single laboratory colonies, temporal instability in microbiome composition has been documented (Terenius et al., 2012). These inconsistencies reflect genuine ecological contingency. The microbiome is a product of host–environment interactions that vary by definition, but they also reflect methodological heterogeneity in DNA extraction protocols, 16S rRNA variable regions amplified, sequencing platforms, and bioinformatic pipelines (Minard et al., 2013). This lack of standardization complicates cross-study comparisons and makes it difficult to identify robust, context-independent relationships between specific microbiome features and vector competence.

### 7.2 Ecological contingency and evolutionary responses

Interventions designed and validated under controlled conditions face the challenge that their effects are mediated through ecological interactions that are inherently contingent. The pathogen-blocking efficacy of *Wolbachia* strains varies with host mosquito genotype, ambient temperature, and the presence of co-infections (Ross et al., 2017; Ye et al., 2015). Temperature increases consistent with future climate change projections have been shown to reduce wMel density in *Ae. aegypti* and partially attenuate pathogen blocking, raising concerns about strategy durability in thermally variable or warming climates (Frentiu, 2017). Evolutionary responses represent a further concern. Sustained selective pressure could favor mosquito genotypes less permissive to symbiont colonization, or viral genotypes with altered susceptibility to *Wolbachia*-mediated blocking. While empirical evidence for such evolutionary escape is limited, the theoretical risk is non-negligible and should be incorporated into long-term monitoring frameworks.

### 7.3 Biosafety, ecological spillover, and regulatory issues

The release of modified microbiomes raises genuine biosafety and ecological concerns. *Wolbachia* strains released in *Ae. aegypti* are maternally transmitted and therefore self-spreading in target populations, meaning that once introgression has occurred, it cannot easily be reversed. For paratransgenic organisms, concerns about the persistence of transgenes in environmental microbial communities are more acute, given high rates of horizontal gene transfer among bacteria. Regulatory frameworks for environmental release of genetically modified organisms vary substantially by jurisdiction and have not yet converged on standardized risk-assessment protocols specifically designed for microbiome-based vector-control interventions (Bourtzis et al., 2016).

### 7.4 Ethical issues and community acceptance

Ethical dimensions extend beyond biosafety to include questions of equity, consent, and community governance. Large-scale releases take place in communities, often in low-income countries, that bear the burden of vector-borne diseases but may have limited institutional capacity to evaluate and monitor the interventions being implemented in their environments. The degree to which meaningful community consent can be obtained for population-level interventions that affect shared public space is a substantive ethical issue not fully resolved in existing frameworks (Otolorin et al., 2026). Transparency about benefits, risks, and uncertainties, combined with genuine partnership with affected communities in trial design and monitoring, is essential for the ethical legitimacy of field programs.

## 8 Emerging technologies and future directions

Table 4, which summarizes the principal emerging omics and systems biology technologies most likely to advance mosquito microbiome science from its current descriptive phase to genuinely predictive and intervention-relevant understanding, is introduced here and cross-referenced throughout this section.

**Table 4:** Priority emerging technologies for advancing mosquito microbiome research from descriptive to predictive science.

Technology	Application in mosquito microbiome research	Current status	Key advancement required
Shotgun metagenomics	Full taxonomic + functional profiling of all microbiome kingdoms simultaneously	Increasingly routine	Standardized bioinformatic pipelines; reference databases for mosquito-specific taxa
Metatranscriptomics	Active gene expression profiling during blood feeding, pathogen challenge, immune activation	Early adoption	Protocols optimized for low-input mosquito material; temporal resolution studies
Metabolomics (LC-MS/GC-MS)	Mapping microbiome-derived metabolites mediating pathogen–host interactions	Growing use	Pairing with metagenomics in same individuals; metabolite library development
Culturomics	High-throughput isolation of previously uncultured mosquito-associated bacteria	Emerging	Automation; growth condition diversification for obligate gut symbionts
Single-cell RNA sequencing	Cell-type-specific mosquito immune transcriptomics during microbiome perturbation (Raddi et al., 2020)	Early research stage	Reference single-cell atlases for An. gambiae and *Ae. aegypti* midgut epithelia
Spatial transcriptomics	Anatomical mapping of microbiome–host interaction sites within mosquito tissues (Vial et al., 2025)	Pre-application	Tissue fixation protocols adapted for small insect organs
Machine learning/AI	Prediction of vector competence from microbiome composition; intervention design optimisation	Early modeling studies	Large, harmonized, geographically diverse training datasets
Long-term field cohort studies	Documenting microbiome temporal dynamics alongside entomological and epidemiological data (Otani et al., 2025; Lee et al., 2024)	Very limited	Multi-year funded longitudinal platforms in high-burden settings

LC-MS, liquid chromatography–mass spectrometry; GC-MS, gas chromatography–mass spectrometry; AI, artificial intelligence.

### 8.1 Advanced omics approaches

The descriptive phase of mosquito microbiome research, dominated by 16S rRNA amplicon sequencing surveys, is increasingly being supplemented by more comprehensive omics platforms. Shotgun metagenomics enables simultaneous characterization of bacterial, fungal, viral, and protozoan microbiome components at the functional gene level, overcoming the taxonomic resolution limits of amplicon approaches and enabling reconstruction of microbial metabolic pathways directly relevant to vector competence (Muturi et al., 2017; Wang et al., 2023). Metatranscriptomics reveals which microbial functions are actually active under different physiological conditions, such as during blood-meal digestion or pathogen challenge, rather than merely which organisms are present (Strand, 2018; Ragini et al., 2025). Culturomics-high-throughput isolation and culture using diverse growth media-expands the repertoire of organisms available for functional characterization and manipulation (Tandina et al., 2016). See Table 4 for a structured overview of each technology's current status and key methodological requirements.

### 8.2 Single-cell, spatial, and systems biology approaches

Single-cell RNA sequencing of mosquito tissues has begun to reveal cell-type-specific immune gene expression responses to microbial colonization. Raddi et al. (2020) provided the first single-cell transcriptomic map of *Anopheles gambiae* cellular immunity, revealing the diversity of immune cell populations responding to midgut microbiota. Vial et al. (2025) applied spatial transcriptomics to *Ae. aegypti* midgut tissues, enabling anatomical mapping of microbiome–host interaction sites that amplicon and bulk RNA-seq approaches cannot resolve. When combined, these approaches can map precisely where in the mosquito body microbiome–host and microbiome–pathogen interactions occur. Machine-learning approaches are increasingly being applied to microbiome datasets to identify compositional signatures associated with vector competence, to predict the outcomes of microbiome perturbation experiments, and to optimize intervention designs. These technologies are referenced in Table 4.

### 8.3 One health and longitudinal field studies

The One Health framework, which recognizes the interdependence of human, animal, and environmental health, provides a productive conceptual context for mosquito microbiome research. Long-term, geographically replicated field studies that characterize microbiome dynamics alongside entomological, epidemiological, and environmental variables are needed to move beyond cross-sectional snapshots toward genuinely predictive understanding of how ecological change modulates vector competence through microbiome pathways. Tokash-Peters et al. (2024) reported one of the first longitudinal mosquito microbiome field studies tracking seasonal dynamics in Rwanda. Otani et al. (2025) documented mosquito-borne bacterial communities shaped by host species, geography, and developmental stage across a multi-year, multi-country European framework, demonstrating that continent-scale standardized longitudinal surveillance is operationally feasible. Akintola and Hwang (2024) and Lee et al. (2024) characterized microbiomes across geographical gradients in South Korea, providing a direct regional context for the South Korean-based research contributing to this review.

## 9 Outstanding research questions

The following questions represent the most important unresolved issues in mosquito microbiome science that should guide future research agendas:

1. What are the minimum functional microbiome requirements for normal mosquito larval development across different Anopheles, Aedes, and Culex species, and do these involve specific bacterial taxa or functional guilds that are transferable across host species?
2. To what extent do field microbiome compositions, which are far more complex than laboratory model systems, mirror or depart from the simplified communities used in most mechanistic experiments, and does this discrepancy systematically bias current estimates of microbiome effects on vector competence?
3. What is the quantitative relationship between Wolbachia tissue density, host genotype, ambient temperature, and pathogen-blocking efficacy across different arbovirus–mosquito combinations, and can this relationship be parameterized sufficiently for predictive epidemiological modeling?
4. How rapidly can mosquito or pathogen populations evolve in response to Wolbachia-imposed or other microbiome-imposed selection pressures, and what monitoring tools are required for early detection of evolutionary rescue or resistance?
5. What delivery systems can maintain ecologically persistent colonization of field Anopheles populations by pathogen-suppressive bacteria, in the absence of maternal inheritance, and at what cost-effectiveness threshold would such systems become competitive with existing interventions?
6. Do insect-specific viruses represent a tractable biocontrol resource against medically important arboviruses, and if so, what delivery mechanisms could be used for their introduction into natural mosquito populations without producing unintended ecological effects?
7. What governance frameworks, regulatory standards, and community-engagement protocols should govern the field release of microbiome-modifying interventions, and how can these be harmonized across high-burden, low-resource countries with differing regulatory capacities?
8. How do climate-driven changes in vector range, breeding-site ecology, and thermal physiology alter mosquito microbiome composition, and can microbiome-based interventions be designed to remain effective across a range of projected future climate scenarios?

## 10 Conclusion

The mosquito microbiome has emerged from relative obscurity to occupy a central position in modern vector biology, and for good reason. Microbial communities associated with mosquitoes are not epiphenomenal passengers but active determinants of host physiology, immunity, and pathogen transmission competence. This review has synthesized evidence demonstrating that the microbiome influences mosquito biology from larval development through to adult fitness and behavior, and that it can substantially alter the capacity of mosquitoes to transmit the most medically important pathogens through diverse immunological, metabolic, and competitive mechanisms.

**Figure 4:**
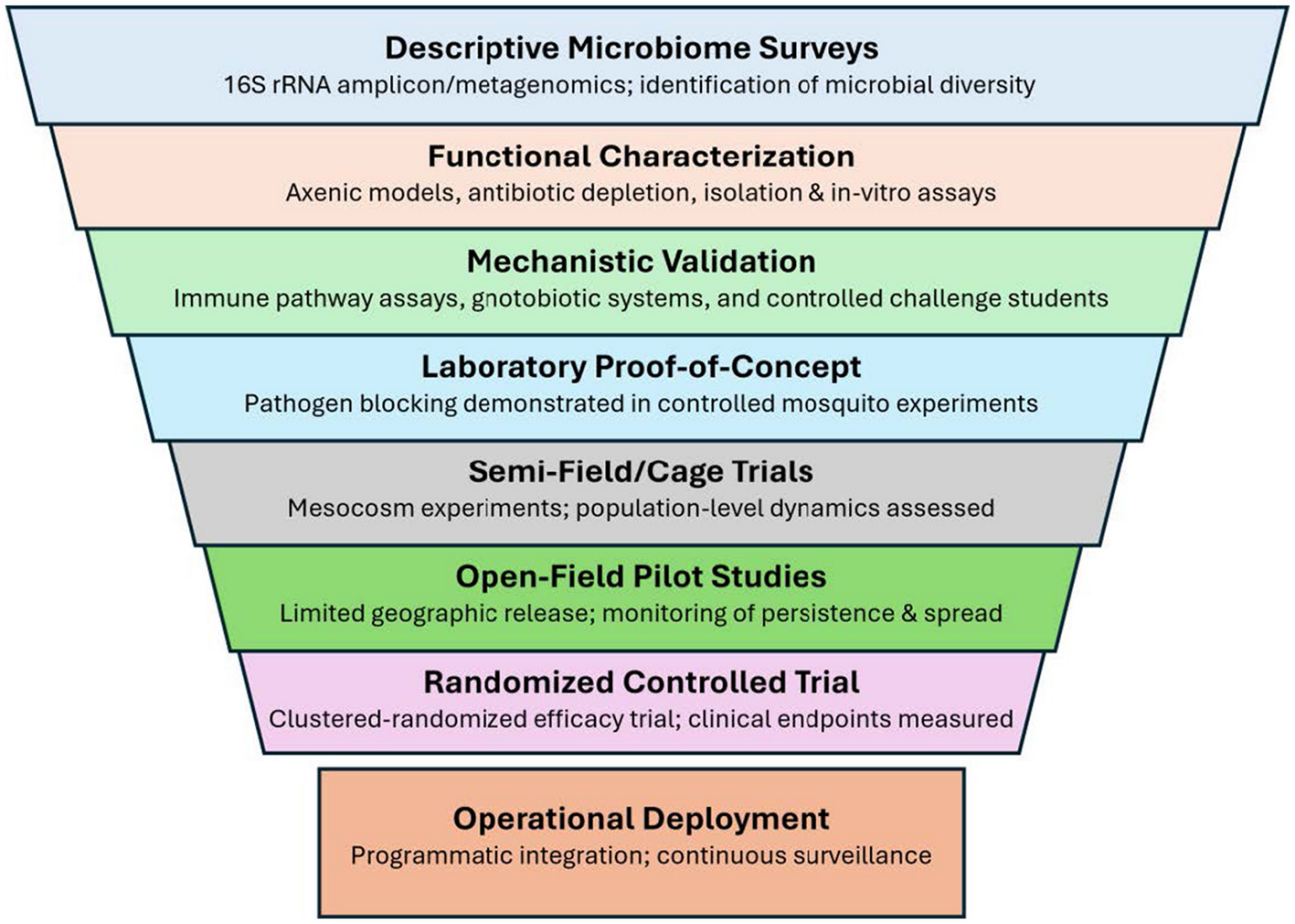
Research-to-implementation funnel for microbiome-based vector-control interventions. The translational funnel illustrates the progressive attrition from basic descriptive microbiome surveys to operational programmatic deployment, with key transition barriers annotated at each stage. *Wolbachia* (wMel in *Ae. aegypti*) is the only strategy to have reached the randomized controlled trial stage. Most alternative strategies remain in early laboratory or proof-of-concept phases. The narrowing funnel width reflects the declining number of interventions that survive each successive validation hurdle.

The strongest and most translatable evidence for microbiome-based disease control comes from *Wolbachia* deployment in *Ae. aegypti*. The randomized controlled trial in Yogyakarta (Utarini et al., 2021) represents a genuine landmark: a microbiome-level biological intervention producing clinically meaningful reductions in dengue incidence in a real-world urban setting — a translational trajectory captured schematically in Figure 4. Other strategies, such as paratransgenesis, bacterial supplementation, insect-specific virus exploitation, and habitat-level microbiome management, remain at earlier stages and face substantial feasibility barriers that have not yet been resolved.

Three priority gaps require urgent attention. First, mechanistic understanding of microbiome–pathogen–vector interactions remain largely grounded in reductive laboratory experiments; the degree to which these findings apply to ecologically complex field settings demands systematic evaluation through standardized longitudinal field studies (Otani et al., 2025; Ragini et al., 2025). Second, unlike *Ae. aegypti* where stable wMel transinfection achieved naturally self-spreading introgression, no analogous strategy has been stably deployed in wild *An. gambiae* populations, representing the central unsolved challenge for microbiome-based malaria control, although wAlbB advances in *An. stephensi* provide cause for cautious optimism (Joshi et al., 2014; Liang et al., 2024). Third, the eukaryotic microbiome, encompassing fungi, protozoa, and nematode-associated organisms, remains substantially undercharacterized relative to its likely functional significance, and kingdom-comprehensive metagenomics approaches are needed to resolve this gap (Hegde et al., 2024; Palinauskas et al., 2024).

In summary, the modulation of the mosquito microbiome represents one of the most biologically innovative and ecologically grounded frontiers in vector-control science. Though promising, it requires sustained interdisciplinary collaboration, rigorous field validation, and genuine engagement with the communities that both stand to benefit most and bear the greatest risk from unintended consequences.
